# Gut microbiome modulation for military resilience and performance

**DOI:** 10.3389/frmbi.2026.1828981

**Published:** 2026-07-24

**Authors:** Emma F. Murphy, Iain Templeman, Joanna Rimmer, Sarah V. Harding

**Affiliations:** 1 Human Biological Advantage Team Defence Science and Technology Laboratory, Salisbury, United Kingdom; 2 Academic Department of Military Medicine, Royal Centre for Defence Medicine, Birmingham, United Kingdom

**Keywords:** gut, military, modulation, performance, regulation

## Abstract

A range of technologies are being developed to modulate the human gut microbiome, aimed at resolving gut dysbiosis and restoring normal host function. Although limited, a subset of studies have begun to evaluate these technologies within healthy human populations. This could provide approaches to mitigate the impact of occupational stressors on military personnel to ensure their operational effectiveness and resilience is maintained, and could also extend to enhancing the physical or cognitive performance of an individual beyond their baseline potential. Research using *in vivo* models and healthy human populations suggest that cognition, mineral absorption, muscle resilience, endurance and structural integrity, and injury recovery are modified by the gut microbiome. However, the regulations that govern the use of these technologies are largely focused on their use in treating disease and promoting health, which could hinder such applications. Therefore, whilst the use of gut microbiome modulation could present opportunities to enhance resilience and performance in military personnel, more research in healthy human cohorts is needed, alongside the development of effective regulatory frameworks supporting wider applications.

## 1 Introduction

The gut microbiome is an ecosystem of interacting microorganisms (‘the microbiota’) which has been shown to influence host biology (Ogunrinola et al., 2020). Within the gut this includes changes to metabolite production, gut barrier integrity, susceptibility to infection, and immunomodulatory activity. Beyond the gut, behavioural and cognitive responses, the response to different stressors and overall wellbeing via the gut-brain axis can also be altered (Allen et al., 2016; Martin and Mayer, 2017). Disturbances to this ecosystem due to illness or physical stress can lead to dysbiosis, disrupting gut homeostasis, and by extension could modify these key dimensions of host biology (Krogius-Kurikka et al., 2009; David et al., 2014; Lloyd-Price et al., 2019).

Diet is a key factor in shaping gut microbiome composition and activity, with correlations being drawn between diet and physical performance of military personnel (Robillard et al., 2026). This can be an issue for Defence where ration packs, an energy deficit and reduced access to fresh food are common challenges. However, diet and operational nutrition are beyond the scope of this review. In addition, deployment relevant environmental stressors have been shown to alter the gut microbiome and cognition of otherwise healthy populations, and highlight potential metabolites linked to physical performance (Karl et al., 2019; Shi et al., 2022; Ma et al., 2025; Liechty et al., 2026). An in-depth overview of Defence-related microbiome research is provided in the ‘Meeting report of the eighth annual Tri-Service Microbiome Consortium Symposium’ (Kardish et al., 2025).

**Figure 1:**
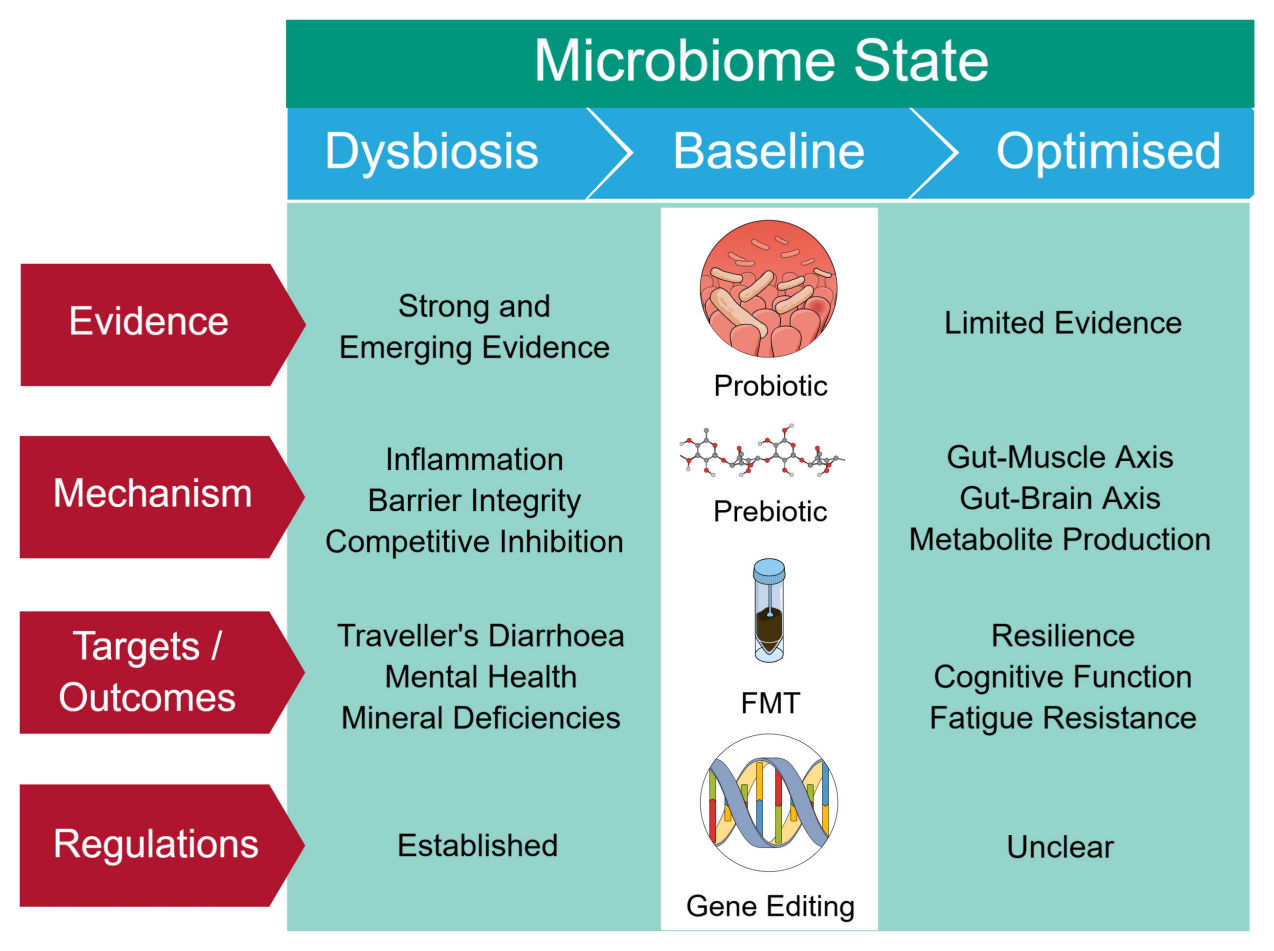
A summary table of the GMMTs described. The table outlines the level of evidence, the mechanism of action (although non-exhaustive), the potential target/outcomes of the modulation, and the current regulatory definitions of the GMMTs included in this review. The summations differ depending on the health status of the microbiome, i.e. dysbiotic (left) and optimised (right) state. Created in https://mindthegraph.com.

Various gut microbiome modulation technologies (GMMTs) have been developed to resolve dysbiosis, protect against disease and promote gut health (**Figure 1**) (Gao et al., 2010; Baunwall et al., 2022; Brödel et al., 2024; Srivastava et al., 2024). However, applications beyond maintaining health are currently limited by understandings of microbe-microbe and host-microbe interactions, and the governing regulatory frameworks. Despite this, emerging studies suggest GMMTs, present an opportunity to augment the gut microbiomes of healthy cohorts to improve resilience and performance. GMMTs could help to preserve personnel host-microbiome interactions in the context of operational stressors i.e. extreme environmental conditions (Allen et al., 2016; Huang et al., 2019; Pugh et al., 2019; Schreiber et al., 2021). This review aims to provide an overview of current GMMTs (probiotics, prebiotics and faecal microbiota transplantation) and introduce emerging GMMTs (gene editing) as potential methods for gut microbiome modulation to enhance resilience and performance in military personnel, whilst considering their feasibility under current regulations.

## 2 Overview of gut microbiome modulation technologies

Dysbiosis is the alteration of microbial composition which can negatively impact host physiology (Buttó and Haller, 2016). Therefore, probiotics are composed of live microorganisms which confer a host benefit, but require routine administration to have a sustained effect (Hill et al., 2014). Once established, these microorganisms can inhibit pathogenic bacterial growth and colonisation, produce antimicrobials, support immune function and produce key metabolites that promote epithelial integrity (Klaenhammer, 1988; Lebeer et al., 2008).

Nutrients are also required for the constituent microbes to survive. Prebiotics are substrates which act as an external source of nutrients for the gut microbiota and are selectively used to confer a host benefit (Davani-Davari et al., 2019). By-products of their fermentation are short-chain fatty acids (SCFAs) which influence downstream host biological processes including cellular structure and signalling, immune and inflammatory regulation, and metabolic and oxidative stress responses (Deehan et al., 2024; Mann et al., 2024). This ultimately lowers the pH of the colon and stimulates commensal and/or probiotic growth to inhibit pathogenic growth and aid intestinal development (Walker et al., 2005; Hamer et al., 2008; Davani-Davari et al., 2019; Cunningham et al., 2021; Deehan et al., 2024).

**Figure 2:**
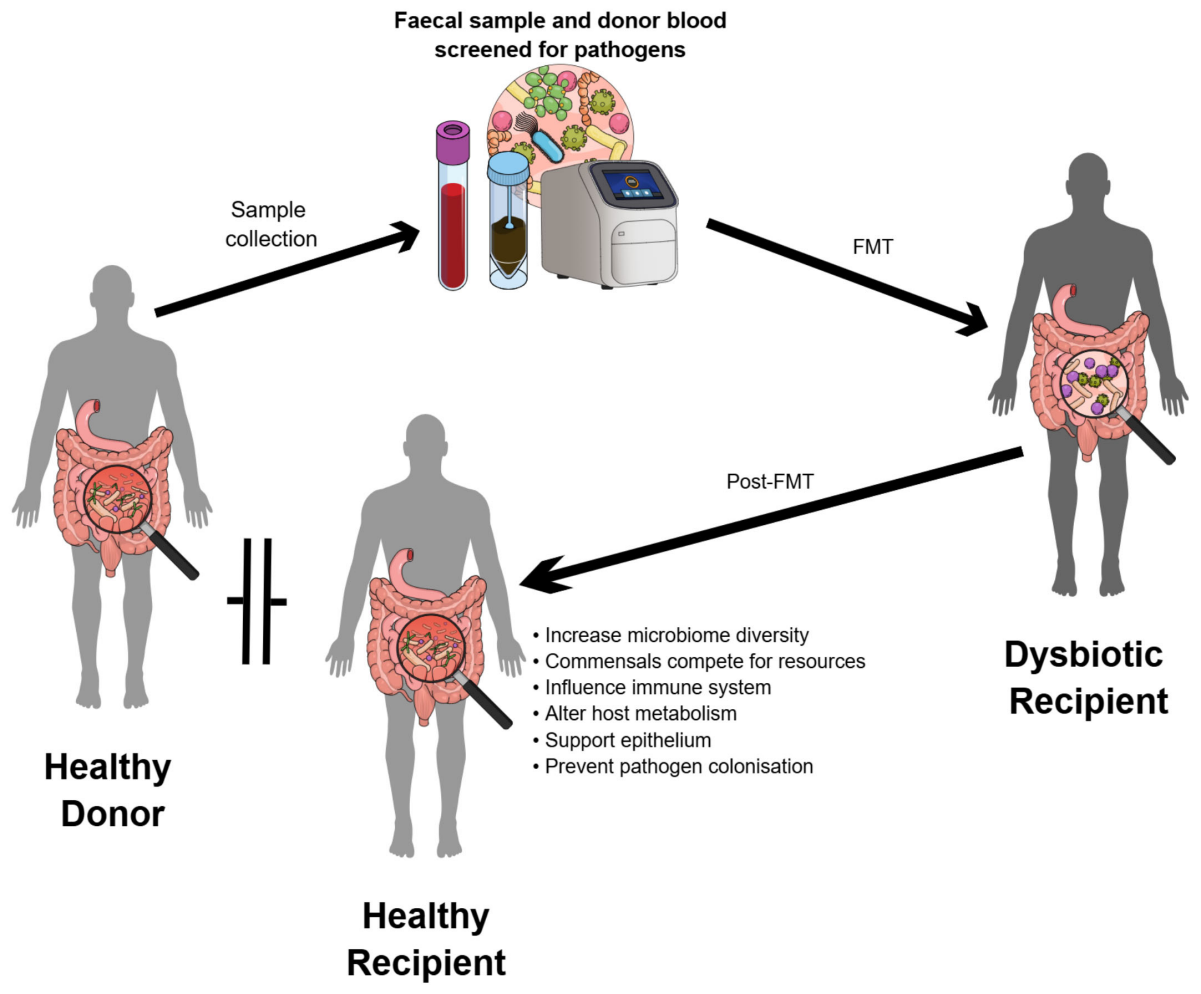
The FMT process. A faecal sample and a blood sample are obtained from a ‘healthy’ donor and screened for pathobiont species before transplantation into the gastrointestinal tract of an ‘unhealthy’ recipient. The bacteria in the ‘healthy’ sample colonise the gut and resolve dysbiosis. Created in https://mindthegraph.com.

The microbiota composition can have a beneficial or detrimental effect on host biology. Therefore, a ‘healthy’ individual’s microbiome can act as a template for colonising the microbiome of others. Faecal microbiota transplantation (FMT) attempts to colonise the gastrointestinal (GI) tract of a dysbiotic recipient with a functioning microbial ecosystem derived from a ‘healthy’ donor (Yadegar et al., 2024) (**Figure 2**). FMT can be delivered by several enteral routes, with preparations in development for oral administration (Gulliver et al., 2022). In the UK, it is only licensed for treating recurrent *Clostridium difficile* infections. Following colonisation, the commensal bacteria from ‘healthy’ donors can outcompete pathogens for nutrients, stimulate the immune system, support intestinal epithelial cell integrity, alter host metabolism and increase microbial diversity (Seekatz et al., 2014; Buffie et al., 2015; Khoruts and Sadowsky, 2016).

Gene editing allows for the precise targeting of microbial genes. This can enable microbiome modulation by: 1) the selective removal of particular genes or bacterial strains, or 2) the modification of commensals to introduce new or optimise exisiting characteristics (Pacesa et al., 2024). Gene editing could be used to refine bacterial strains in GMMTs. Examples of this include the development of a probiotic with increased metabolite production *in vitro* or by targeting a pathogen *in vivo* to increase specificity of killing (Desmond et al., 2004; Goh and Barrangou, 2019; Ren et al., 2022). However, current limitations to these approaches include feasibility, ethics, and translational readiness in the human gut, particularly in military cohorts.

## 3 The use of gut microbiome modulation technologies for resilience and performance

The associations between the gut microbiome and various health- and disease-related outcomes have resulted in the development of various products for health improvement applications. There is also interest in how the gut microbiome could be modulated to enhance human resilience and physical and cognitive performance (Shing et al., 2014; Allen et al., 2016; Huang et al., 2019; Papalini et al., 2019). Relatively few studies have considered GMMTs for wider applications. However, some have explored GMMTs, such as probiotics, for resilience and performance.

Stress and sleep deprivation have been linked to deteriorations in cognition and memory (Alhola and Polo-Kantola, 2007; Kim and Kim, 2023). One study showed that intervention with *Bifidobacteria longum* 1714 reduced cortisol levels, self-reported anxiety and stress, and improved memory in 22 healthy adults (Allen et al., 2016). A similar study found that multispecies probiotics (containing lactobacilli and *Bifidobacterium*) protected against memory loss induced by stress (Papalini et al., 2019). Probiotics may therefore provide an opportunity to reduce the impact of occupational stress and sleep deprivation on cognitive function within military cohorts.

These strategies have also been investigated to improve the physicality of an individual. A double-blind placebo-controlled study suggests that supplementation with *Lactobacillus plantarum* can improve exhaustion time, reduce biomarkers of fatigue, accelerate recovery and increase muscle mass (Huang et al., 2019). In two separate studies, probiotics were found to increase time to fatigue, and reduce inflammation and oxidative stress arising from muscle fatigue in athletes (Martarelli et al., 2011; Shing et al., 2014). An *in vivo* study demonstrated that the probiotic *Lactobacillus rhamnosus* could upregulate antioxidant enzymatic pathways during periods of physical exercise to reduce muscle fatigue and increase endurance (Yang et al., 2024). Whilst another *in vivo* study explored the effects of FMT on physical fitness in young and old mice. They observed a link between gut microbiome composition, gut barrier leakiness, frailty (measured as grip strength), physical activity and running speed, memory, muscle atrophy and inflammation (Zhu et al., 2024). These studies suggest that GMMTs may be utilised to influence physical performance to combat changes associated with factors like physical exhaustion and aging, however, this requires further investigation in larger human cohorts.

During periods of intense exercise and physical stress, an increased risk of GI upset and infection has been correlated with factors including increased intestinal permeability and altered immune function due to changes in gut microbiome composition (Allen et al., 2015; Gleeson and Pyne, 2016; Karl et al., 2017). A study investigating the use of probiotics in 27 elite male cyclists reported a reduction in GI issues during training by 27% compared to an 8% increase in the placebo group (Schreiber et al., 2021). A similar study also reported a reduction in GI symptoms in marathon runners during the final third of a race when using probiotics compared to the placebo group (Pugh et al., 2019). In addition, the use of probiotics in elite athletes was correlated with a reduction in the duration of upper respiratory tract infections by 21.5%, the increased production of adaptive immune cells and a higher level of saliva IgA (de Vrese et al., 2005; Gleeson et al., 2011). Probiotics may therefore help to protect the body against disruptions associated with intense physical stress.

## 4 Relevance to defence

Military personnel are routinely exposed to different occupational stressors, including extreme environmental conditions, prolonged periods of physically demanding tasks and exposure to endemic pathogens which can impact their readiness and performance (Connor and Gutierrez, 2013a; Gepner et al., 2017; Sun et al., 2022). The studies described above could therefore present a number of valuable opportunities. Enhancing performance can be defined as ‘changing the baseline of an individual beyond their normal state or by helping an individual to maintain their baseline level of performance,’ which would otherwise be reduced following exposure to a stress/stressors. Subsequently, GMMTs to enhance performance may support the resilience of military personnel.

Clinical studies have investigated the use of prebiotics, including inulin, to increase mineral absorption, e.g. calcium. A double-blind placebo-controlled trial identified a 5.1% increase in calcium absorption and a 5.2% increase in magnesium absorption following consumption of inulin over 6-weeks in menopausal women (Holloway et al., 2007). Another study reported similar findings in young healthy adults, demonstrating a greater than 3% increase in calcium absorption predominantly within the colon, potentially due to altered cellular signalling enabling the passive absorption of minerals and an increased intestinal surface area (Abrams et al., 2007). The enhanced absorption of minerals may benefit defence when treating mineral deficient related pathologies, including vitamin disorders, often associated with military deployments and musculoskeletal injuries (Farina et al., 2017; Knapik et al., 2021; Pav et al., 2024; Lovalekar et al., 2025).

Across all services, military personnel risk obtaining traumatic brain injuries (TBIs), and a link between traumatic head injury and subsequent gut microbiome dysbiosis has been reported (Treangen et al., 2018). An *in vivo* study investigating the use of prebiotics to support the treatment of TBIs, observed changes in microbial composition and diversity which correlated with the protection of brain tissue and cerebral blood flow (Yanckello et al., 2022). It was proposed that SCFAs helped to mitigate neuroinflammation, reducing tissue damage post-injury (Yanckello et al., 2022). Prebiotics may therefore be utilised to support rehabilitation in the future.

The extreme conditions experienced by submariners can lead to the development of “seafaring syndrome” (abnormal defecation frequency, insomnia, poor sleep quality, nausea and overeating) (Sun et al., 2022). Metagenomic analysis has shown that its development is associated with changes in microbial signatures similar to those associated with chronic disease, including neurological, metabolic and autoimmune disease (Jiang et al., 2022; Sun et al., 2022). A longitudinal placebo-controlled study showed that following administration of probiotics in 82 sailors their microbial signature and functional composition were maintained over 30 days (Zhang et al., 2020). There was also a greater presence of commensal species including *Bifidobacteria* and a reduction in self-reported anxiety and stress (Zhang et al., 2020). The use of such products could support cognitive resilience in submariners.

During sustained military operations, the ability to complete physical tasks requiring the use of muscle strength can be reduced (Nindl et al., 2002). A randomised placebo-controlled study investigated the use of *Bacillus coagulans* to increase the absorption of a metabolite known to attenuate muscle damage during high-intensity exercise (Gepner et al., 2017). Their combined administration was found to reduce inflammatory responses and alter muscle diffusion coefficients in 18 soldiers undergoing intense military training, suggesting increased muscle protection (Gepner et al., 2017). Enhancing absorption of functional metabolites using probiotics may help protect the muscle integrity of military personnel on sustained operations.

Operational activity in a deployed environment can lead to an increased exposure to endemic pathogens and a reduction in workforce effectiveness as a result of developing Travellers’ Diarrhoea (Connor and Gutierrez, 2013a; Troth et al., 2023). During the Afghanistan conflict, the British Military experienced a 40% diarrhoeal disease rate over a 6 month period with an estimated 46,256 duty-days lost (Connor et al., 2013). Gurkha soldiers have been observed to have a lower incidence of Traveller’s Diarrhoea than non-Gurkha British soldiers (Connor and Gutierrez, 2013b). This resistance could be associated with differences identified in the microbial diversity of collected faecal samples (n=38) at the species and genus level (Troth et al., 2023). GMMTs could therefore be utilised to reduce infections, including Travellers’ Diarrhoea, by supporting the growth of commensal bacteria that functionally protect against diarrhoeal pathogens. Further research is needed in this area.

The analysis of nutrient-related gene expression in space using integrated omics highlighted that the environment can lead to the disruption of metabolic pathways resulting in vitamin-related disorders, particularly in those with genetic predispositions (Barbero Barcenilla et al., 2025). Gene editing, combined with pharmacogenomics, could be applied to better determine the metabolic pathways effected by military occupational stressors and optimise or even personalise GMMTs, like probiotics, through the refinement of bacterial strains for increased killing, metabolite production and absorption as previously described.

## 5 The regulatory landscape

The regulatory landscape governing microbiome modulation approaches is highly complex, as different countries have their own regulators who define the technology and regulate it differently. This is further complicated when considering these technologies beyond their intended use – typically to resolve dysbiosis and disease. In the context of resilience and performance, some approaches if used to enhance performance above baseline do not align with this definition, but do align if used to help maintain this baseline during periods of stress. Such nuances present barriers to the wider exploitation of microbiome modulation techniques.

In the UK, probiotics are classed as live biotherapeutics and are considered to be “a product presented for treating or preventing disease, which may be administered with a view to restoring, correcting or modifying physiological function in humans”. Accordingly, they are regulated by the Medicines and Healthcare Products Regulatory Agency (MHRA) (DHSC, D.o.H.S.C 2021b; MHRA, M.H.P.R.A, 2022). Probiotic foods and dietary supplements, however, are regulated by the UK Nutrition and Health Claims Committee (UKNHCC) under the Department of Health and Social Care (DHSC). Only one multi-strain probiotic has been approved in Europe for lactose maldigestion by the European Food Safety Authority (EFSA, E.F.S.A, 2010). The use of probiotics to modify physiological function beyond the baseline of an individual is not currently permitted.

There are no approved prebiotics authorised under the Commission Regulation (EU) 432/2012 of 16/05/2012 (Great Britain Nutrition and Health Claims Register) (DHSC, D.o.H.S.C, 2020). For prebiotics, any health claim submission including reference to psychological and behavioural functions, or that states, suggests or implies an ability to reduce the risk of developing a disease, is covered under nutritional legislation and assessed by the UKNHCC (DHSC, D.o.H.a.S.C, 2021a). Similar to probiotics, modifying physiological function beyond baseline does not fit the current regulatory definition.

FMT is classified as a medicinal product by the MHRA (MHRA, M.H.P.R.A, 2020). A medicinal product is defined as “any substance or combination of substances presented as having properties for treating or preventing disease in human beings” (MHRA, 2012). Again, this would not permit the use of FMT to enhance performance beyond baseline. Larger data sets demonstrating the use of FMT to modulate other host biological processes would be required to demonstrate its ability to be licensed for alternative purposes.

Gene editing is highly restricted and has only recently been licensed in the UK for use in humans (NICE, 2025). Genetically edited probiotics to prevent disease or maintain health to protect performance levels could be classified as a gene therapy product under the MHRA (MHRA, M.H.P.R.A, 2025). However, when used to modulate the gut microbiome to enhance performance, gene editing may be better classified as a gene driver regulated by the Department of Environmental, Food and Rural Affairs (Mir et al., 2022).

Further updates to the current regulatory definitions are required to ensure there are effective regulations in place for microbiome modulation beyond the treatment or prevention of disease. This will require further studies to show the benefit of these approaches for those additional applications.

## 6 Discussion

There are several scenarios within defence where occupational stressors may impact personnel performance (Nindl et al., 2002; Connor and Gutierrez, 2013a; Farina et al., 2017; Knapik et al., 2021; Sun et al., 2022). Research exploring gut microbiome modulation in the context of health and disease has identified various pathways which could be used to enhance human resilience and performance.

The data generated from clinical studies investigating the use of GMMTs are limited often due to small cohorts and therefore it is difficult to make definitive conclusions (Ladirat et al., 2014; Kang et al., 2017; Liu et al., 2021). Larger sample sizes, particularly in non-clinical cohorts, are required to increase data confidence and to enable stronger conclusions to be made regarding their use in military personnel. In addition, the studies performed to date typically involve young male cohorts, introducing population bias, and do not consider the effects of sex or age, further limiting interpretation of findings.

It must be noted that the selection of the most appropriate GMMT for defence application would depend on the user requirements, the environment/task and the user willingness to adopt these approaches. For example, the use of probiotics and prebiotics may not be appropriate for personnel in an austere conflict environment due to the regularity of administration required to elicit a benefit, but it may be easier to administer in a training environment. The use of gene editing to create genetically engineered probiotics could help to address potential issues associated with storage (and stability) or optimise their effects. This can be demonstrated by the genetically engineered probiotic containing *Escherichia coli* Nissle 1917, designed to release a continuous dose of the creatine precursor, guanidinoacetic acid, for improved cognitive functioning in sleep deprived war fighters (Fields et al., 2025).

Even with the generation of robust scientific data it would be challenging to license such products and few have achieved approval for ‘valid’ health claims (EFSA, E.F.S.A, 2010; NICE, 2025). This is due to a number of issues including the current definitions used and a difficult to navigate regulatory landscape. Changes to the legislation are desirable to ensure that licensing of GMMTs can be effectively regulated for all future use cases.

Utilising GMMTs to augment host function which contribute to enhanced performance may be of use to prevent a reduction in operational effectiveness of military personnel exposed to occupational stressors. Research developing GMMTs in the health and disease industry provides a starting point for achieving this.
